# Claudin-Low Breast Cancer Inflammatory Signatures Support Polarization of M1-Like Macrophages with Protumoral Activity

**DOI:** 10.3390/cancers13092248

**Published:** 2021-05-07

**Authors:** Mayra Cecilia Suárez-Arriaga, Alfonso Méndez-Tenorio, Vadim Pérez-Koldenkova, Ezequiel M. Fuentes-Pananá

**Affiliations:** 1 Unidad de Investigación en Virología y Cáncer, Hospital Infantil de México Federico Gómez, Mexico City 06720, Mexico; msuareza0900@alumno.ipn.mx; 2 Laboratorio de Biotecnología y Bioinformática Genómica, Escuela Nacional de Ciencias Biológicas, Instituto Politécnico Nacional, Mexico City 11340, Mexico; amendezt@ipn.mx; 3 Laboratorio Nacional de Microscopía Avanzada, Centro Médico Nacional Siglo XXI, Instituto Mexicano del Seguro Social, Mexico City 06720, Mexico; vadim.perez@imss.gob.mx

**Keywords:** M1-like macrophages, protumoral activity, breast cancer, claudin-low, tumor microenvironment

## Abstract

**Simple Summary:**

Triple-negative breast cancer (BRCA) cells overexpress the cytokines GM-CSF, G-CSF, MCP-1, and RANTES. We have previously reported that monocytes in 3-D co-culture with BRCA cells generate M1-like macrophages with the ability to induce aggressive features in luminal BRCA cells. Here, we stimulated peripheral blood monocytes with the four cytokines, confirming their capacity to generate protumoral M1-like macrophages. We used the BRCA database to generate an M1-like macrophage gene expression signature related to these cytokines. We observed that the M1-like macrophage, Th1, and immunosuppressive signatures all coincide in claudin-low BRCA but also in mesenchymal carcinomas of colon (COAD) and bladder (BLCA), where they are associated with decreased overall survival in patients. Claudin-low is a tumor subtype with an adverse clinical outcome that remains poorly understood. This study indicates that M1 macrophages may be potential protumoral drivers in already established cancers, and may contribute to the aggressiveness and poor prognosis of claudin-low tumors. These results add to the knowledge of the claudin-low tumor microenvironment and could open a window to immunotherapy strategies to improve patient prognosis.

**Abstract:**

We previously reported that triple-negative breast cancer (BRCA) cells overexpress the cytokines GM-CSF, G-CSF, MCP-1, and RANTES, and when monocytes were 3-D co-cultured with them, M1-like macrophages were generated with the ability to induce aggressive features in luminal BRCA cell lines. These include upregulation of mesenchymal and stemness markers and invasion. In this study, we stimulated peripheral blood monocytes with the four cytokines and confirmed their capacity to generate protumoral M1-like macrophages. Using the METABRIC BRCA database, we observed that GM-CSF, MCP-1, and RANTES are associated with triple-negative BRCA and reduced overall survival, particularly in patients under 55 years of age. We propose an extended M1-like macrophage proinflammatory signature connected with these three cytokines. We found that the extended M1-like macrophage signature coexists with monocyte/macrophage, Th1 immune response, and immunosuppressive signatures, and all are enriched in claudin-low BRCA samples, and correlate with reduced patient overall survival. Furthermore, we observed that all these signatures are also present in mesenchymal carcinomas of the colon (COAD) and bladder (BLCA). The claudin-low tumor subtype has an adverse clinical outcome and remains poorly understood. This study places M1 macrophages as potential protumoral drivers in already established cancers, and as potential contributors to claudin-low aggressiveness and poor prognosis.

## 1. Introduction

Breast cancer (BRCA) has the highest incidence and mortality of all cancers in women of reproductive age worldwide [1]. Different systems of classification have been implemented in the clinic to improve disease prognosis and optimize treatment decision-making. Among the most common are TNM (tumor size, lymph node involvement, and metastasis) staging, and molecular classification based on the expression of estrogen, progesterone, and human epidermal growth factor (Her2) receptors. According to the latter system, BRCA tumors are currently classified into six subtypes: normal-like, luminal A, luminal B, Her2-enriched, basal, and claudin-low. The latter two also referred as triple negative because they lack receptor expression. Although these classification systems have increased the rate of favorable clinical outcomes, tumors often do not respond to standard therapy, or relapse, despite being classified as having a good prognosis [2,3,4,5].

The tumor microenvironment (TME) is defined as the cellular and non-cellular components of a tumor other than the cancer cells. The TME consists of the extracellular matrix and different cell populations (mainly innate and adaptive immune cells). The TME is recognized as a critical factor behind tumor progression [6,7,8]. In recent years, direct targeting of TME elements has shown great promise in the treatment of various unresectable and/or metastatic cancers [9,10,11]. One of the newest therapeutic options is immunotherapy, which aims to enhance the control that the immune system exerts over tumors. However, most BRCA and certain other tumor types do not show objective responses to current immunotherapy [12,13]. A better understanding of the composition of the TME and the molecular and cellular mechanisms by which the TME cooperates or antagonizes tumor progression will undoubtedly lead to better immunotherapeutic strategies [14].

Macrophages are innate immune cells that are essential for the physiological development of the mammary gland, as they secrete cytokines, growth factors, and matrix remodeling enzymes [15]. During estrus/the menstrual cycle, macrophages populate the mammary tissue in response to the ovarian hormones, and the evolution/involution that follows is critically influenced by macrophage function [16,17,18]. *M-CSF*-knockout or sublethally irradiated mice, in which bone marrow-derived monocytes cannot exit, show defective mammary gland development [19,20]. Up to 50% of the total TME cells in BRCA tumors are tumor-associated macrophages (TAMs) [21]. A large TAM infiltration correlates with poor prognosis in BRCA [22] and in other tumor types [23]. TAMs facilitate tumor progression and metastasis by inducing migration, invasion, epithelial-to-mesenchymal transition (EMT), and stem-like properties in cancer cells, as well as angiogenesis and immunosuppression in the TME [24,25,26,27,28,29,30,31,32]. The ability of TAMs to promote stemness is critical for the normal development of the mammary gland, but it also facilitates tumor metastasis, resistance to chemotherapy, and relapse [33].

Macrophages are multifunctional cells that are involved in the elimination of invading pathogens, cancer, and aging cells, as well as in the remodeling/repair of damaged tissue [34]. In spite of this plasticity, macrophages have been traditionally studied as a binary population, enclosing a great variety of functions in only two different subtypes, M1 and M2. The former are credited with proinflammatory and antitumoral functions, and the latter with the TAM activities that facilitate cancer progression and aggressive cancer behavior. This simplistic characterization does not account for the evidence that inflammation is pivotal to the initiation and progression of cancer.

We previously used pro- or anti-inflammatory stimuli to differentiate and polarize monocytes into M1- and M2-like macrophages and found that they more often co-express a mix of M1 and M2 classical markers. In our hands, the markers that best split between M1 (inflammatory) and M2 (anti-inflammatory) stimuli were IL-8 and EGF, respectively [35]. Additionally, when we co-cultured monocytes with three-dimensional (3-D) organoids made of triple-negative BRCA cells (both commercial cell lines and primary isolates), we observed formation of IL-8^pos^ EGF^neg^ macrophages, similar to those formed with proinflammatory (M1) stimuli [3]. These M1-like macrophages promoted extracellular matrix degradation and EMT and invasion in non-aggressive luminal BRCA cells [3,35,36]. Additionally, BRCA cells that could induce macrophages with this activity secreted higher levels of the proinflammatory cytokines GM-CSF, G-CSF, MCP-1, and RANTES than those unable to do so.

To better understand the role of these proinflammatory cytokines in promoting the formation of protumoral macrophages, in this study, we used recombinant cytokines to differentiate peripheral blood monocytes (PBMs) into macrophages. We corroborated the formation of IL-8^pos^ EGF^neg^ M1-like macrophages with protumoral activity. Using the METABRIC BRCA database, we observed that GM-CSF, MCP-1, and RANTES are preferentially expressed in neoplasms with an adverse clinical outcome. We propose an extended M1-like macrophage proinflammatory signature connected with these three cytokines. This signature coexists with monocyte/macrophage, Th1 immune response, and immunosuppressive signatures, and all are enriched in claudin-low BRCA samples as well as in mesenchymal carcinomas of the colon (COAD) and bladder (BLCA).

## 2. Results

### 2.1. GM-CSF, G-CSF, MCP1, and RANTES Drive Formation of M1-Like Macrophages

**Figure 1:**
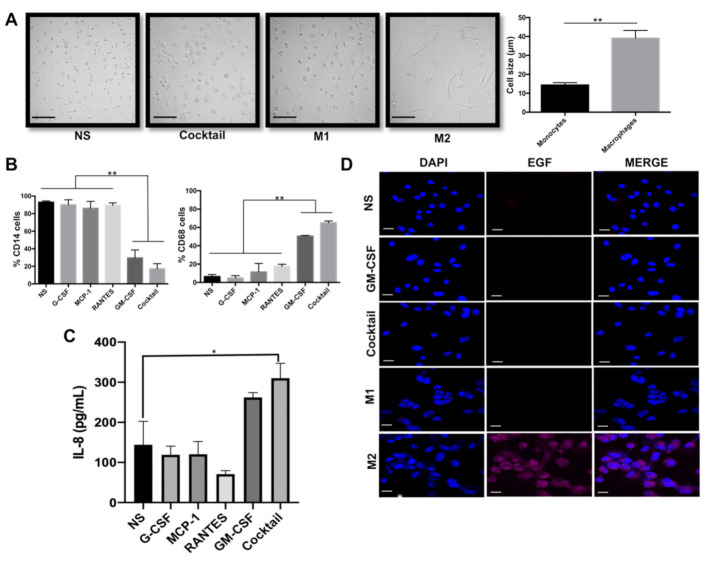
GM-CSF, G-CSF, MCP1, and RANTES promote peripheral blood monocytes (PBMs) differentiation into M1-like macrophages. PBMs were treated with the four cytokines individually or with a cocktail of all of them for six days. (**A**) Monocyte and macrophage morphology (left) and size plot (right) obtained without (NS) or with stimulation with the four cytokines, respectively. The scale bars indicate 100 μm, magnification 100×. (**B**) Flow cytometry of CD14 and CD68 expression. (**C**) IL-8 expression was determined by sandwich ELISA. (**D**) EGF expression was evaluated by immunofluorescence (EGF (magenta) and nuclei (blue)). The scale bars indicate 20 μm, magnification 60×. Macrophages induced with either GM-CSF/LPS/IFNγ (M1) or M-CSF/IL-4/IL-13 (M2) were included for comparison. NS are the non-stimulated PBM cultured six days with medium and 6% FBS. Data represent the mean ± SEM (standard error of the mean) from three independents experiments. Un unpaired Student’s *t*-test was used in (A) ** *p* < 0.01, and a non-parametric Kruskal–Wallis and Dunnett post-hoc test in (B & C) * *p* < 0.05, ** *p* < 0.01.

We have previously shown that supernatants derived from triple-negative BRCA are enriched in the cytokines GM-CSF, G-CSF, MCP-1, and RANTES [3,35,37]. We evaluated whether these cytokines, used at the same concentration found in the BRCA cell supernatants, were capable of inducing PBM isolated from healthy donors to differentiate into macrophages. We observed formation of macrophage-like cells after six days of culture only in PBM treated with GM-CSF alone or with a cocktail of all four cytokines. As shown in Figure 1A and Figure S1A, stimulated cells became large (39 ± 5.17 µm) and adherent, with abundant and vacuolated cytoplasm while non-stimulated control cells or cells stimulated with G-CSF, MCP-1 or RANTES alone maintained the morphology of monocytes (spherical cells growing in suspension, of small size (14 ± 1.4 µm)). Figure 1A shows examples of cells derived with the cytokine cocktail (macrophages) and non-stimulated cells (monocytes). We confirmed preferential expression of CD14 in those cells that retained monocyte morphology, and of the pan-macrophage marker CD68 in the macrophage-like cells (Figure 1B and Figure S1A). We confirmed that the recombinant MCP-1, G-CSF, and RANTES cytokines we used were fully functional, taking advantage of their chemotactic capacity in migration assays, and observing chemotaxis of monocytes U937 with the three cytokines (Figure S1B).

We thereafter determined whether the induced macrophages were polarized into M1-like or M2-like subtypes. For this, we evaluated the expression of IL-8 and EGF and also of the other frequently reported M1 and M2 markers CD86 (for M1) and CD163 and CD206 (for M2). We observed that cocktail-induced macrophages were IL-8^pos^ EGF^neg^ CD86^pos^ but also CD206^pos^, confirming co-expression of M2 markers (Figure S1C); similar results were reported by Chimal-Ramírez [35]. We also induced macrophage polarization using the stimuli GM-CSF/LPS/IFNγ for M1 and M-CSF/IL-4/IL-13 for M2, observing preferential expression of IL-8 and CD86 with the former and of EGF with the latter. The M2 marker CD63 was equally expressed after both stimuli (Figure 1 and Figure S1). We concluded that the cocktail of recombinant cytokines was able to form IL-8^pos^ EGF^neg^ M1-like macrophages similar to the ones that we previously observed in 3-D co-cultures with triple-negative BRCA cells (Figure 1C,D) [3,35,37].

### 2.2. Induced-M1-Like Macrophages Promote Aggressive Features in Breast Cancer Cells

**Figure 2:**
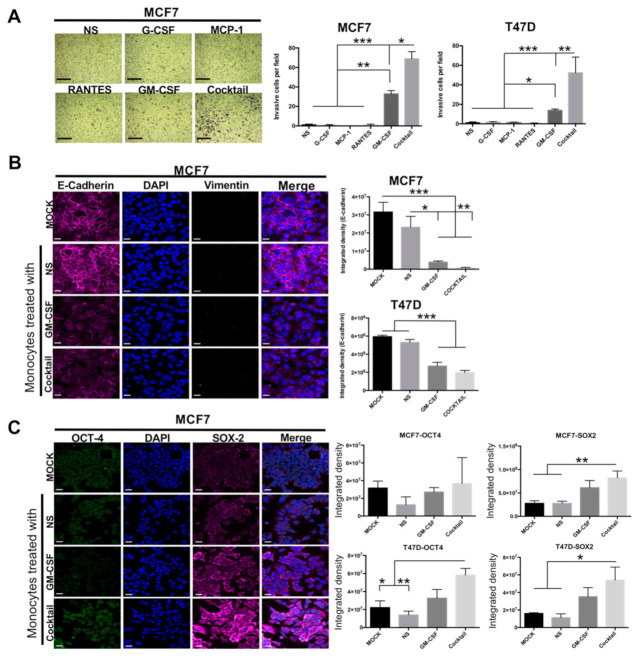
M1-like macrophages induce aggressive features in luminal breast cancer cells. Luminal BRCA cells were co-cultured with cytokine-induced monocytes and controls. (**A**) Matrigel-based transwell invasion assay showing a representative picture (left) and plots (right) of invasive cells per field. The scale bars indicate 100 μm, magnification 100×. Immunofluorescence analysis of EMT (**B**) and stemness (**C**) markers in luminal BRCA cells. (**B**) E-cadherin (magenta), vimentin (green), and nuclei (blue); (**C**) OCT4 (green), SOX2 (magenta), and nuclei (blue). MCF7 representative images are shown (left) and integrated density plots (right). T47D images are shown in Figure S2, and positive staining for vimentin expression in triple-negative MDA-MB-231 cells is shown in S2C. Controls: mock, cells cultured in serum-free DMEM/F12 medium; NS: cells co-cultured with non-stimulated monocytes kept in culture for as many days as the cytokine-induced monocytes. The scale bars indicate 20 μm, magnification 60×. Data represent the mean ± SEM (standard error of the mean) from three independents experiments; non-parametric Kruskal–Wallis and Dunnett post-hoc test * *p* < 0.05, ** *p* < 0.01 and *** *p* < 0.001.

M1 macrophages have been largely considered antitumoral immune cells [38,39,40,41,42]. We have shown previously that triple-negative BRCA cells generated M1-like macrophages that displayed tumor-cooperating mechanisms [3,35,37]. To investigate whether the macrophages polarized with the proinflammatory cytokines also displayed these functions, we evaluated their capacity to coax aggressive features into luminal BRCA cells. In our previous work, we defined the triple-negative BRCA cells as aggressive because of their expression of stemness transcription factors (OCT4 and SOX2) and a mesenchymal marker (vimentin) and lack of expression of an epithelial marker (E-cadherin), and because they have invasive capacity [37,43,44]. We performed invasion assays with MCF7 and T47D luminal BRCA cells in co-culture with non-stimulated or cytokine-induced M1-like macrophages. We observed that GM-CSF- and cocktail-generated M1-like macrophages promoted invasion of the luminal BRCA cells (Figure 2A and Figure S2A), with M1-like macrophages formed with the cytokine cocktail exhibiting the highest efficiency compared with those induced by any of the cytokines alone (with an average of 68 and 52 invasive MCF7 and T47D cells per field, respectively). We then evaluated the induction of EMT and stemness markers by immunofluorescence imaging. We observed a partial EMT phenotype, with a significant decrease of E-cadherin expression but without an increase of vimentin (Figure 2B). We also observed increased expression of OCT4 and SOX2, particularly in M1-like macrophages induced with the cytokine cocktail (Figure 2C). Similar results were obtained with both MCF7 and T47D luminal BRCA cells. Images of experiments performed on T47D cells are shown in Figure S2A–C, and a positive control for vimentin expression is shown in Figure S2D. These data provide further evidence for the capacity of M1-like macrophages to promote features associated with aggressive tumors.

### 2.3. GM-CSF, MCP1, and RANTES Are Overexpressed in Breast Tumors of Poor Prognosis

We explored the METABRIC database to assess the frequency of BRCA expressing the four cytokines, and whether these cytokines are present in tumor subtypes associated with an unfavorable prognosis. The analysis was performed on a cohort of 1221 patients with microarray data and with tumors classified into the luminal, Her2-enriched, and basal intrinsic subtypes (according to the PAM50 profiling test). We downloaded expression and clinical data from METABRIC using cBioPortal. Analyses were performed using the normalized z-score of the microarray data, and genes with a z-score > 1 were defined as overexpressed.

**Figure 3:**
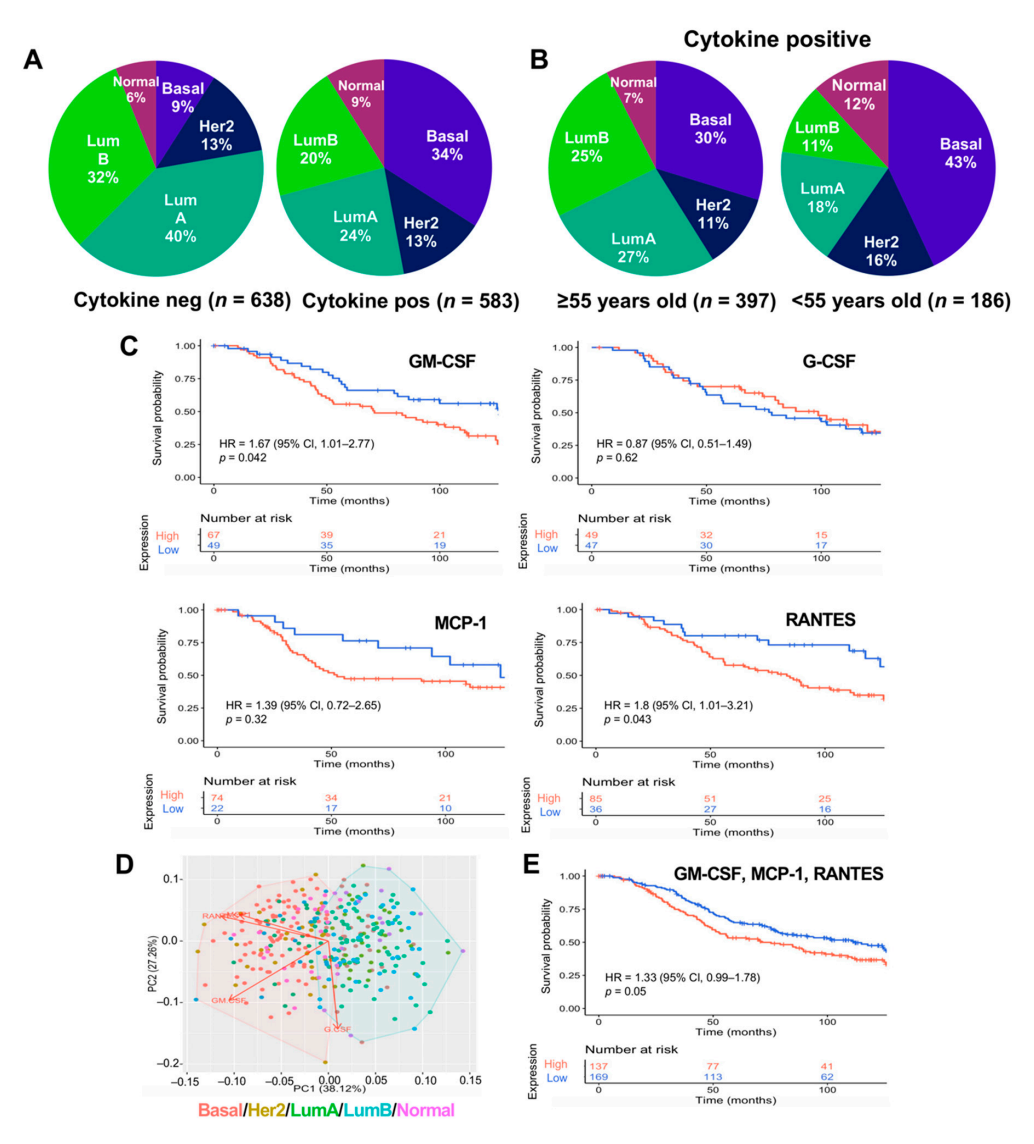
GM-CSF, MCP1, and RANTES are associated with breast cancer aggressive intrinsic subtypes and poor survival in patients above and below 55 years of age. (**A**) Proportion of patients overexpressing any of the cytokines by subtype. (**B**) Proportion of cytokine-positive patients as in A but with patients divided by age, ≥55 and <55. (**C**) Kaplan–Meier survival curves of patients (younger than 55 years old) with GM-CSF, G-CSF, MCP1, or RANTES overexpression. (**D**) Principal component and K-means cluster analyses considering data for the intrinsic subtypes and the four cytokines over-expression. Each subtype is represented by a different color, the clusters are represented in red (aggressive: basal and Her2-enriched) and blue (non-aggressive: luminal and normal) and arrows represent the loadings of the cytokines to PC1 and PC2. (**E**) Kaplan–Meier curves of BRCA patients under 55 years of age with GM-CSF, MCP1, and RANTES overexpression. A sample overexpressing one cytokine was considered when the cytokine showed a z-score >1. Pie charts compare overexpressing samples (cytokine pos) against the rest of the cohort (cytokine neg), while survival curves compare overexpressing (high) vs. underexpressing (low, z-score < −1) samples. Survival curves are a 10-year follow up. *p*-values were calculated using Log-Rank test and the hazard ratios (HRs) were determined with 95% confidence intervals (95% CI).

We first assessed the proportion of samples positive and negative for overexpression of any of the four cytokines by subtype (Table S1). We found the basal subtype was highly represented in the cytokine-positive samples (34%), and poorly represented (9%) in the cytokine-negative samples. We observed the opposite for the luminal subtypes, with a decrease from 40% to 24% in luminal A, and 32% to 20% in luminal B in cytokine-negative and -positive samples, respectively (Figure 3A). BRCA in young women is also known to exhibit more aggressive behavior, with the basal and Her2-enriched subtypes being more common in this population [45,46]. We split the samples with cytokine overexpression in two groups, <55 and ≥55 years of age, and determined the proportion of BRCA subtypes in both age groups. We observed a further enrichment in the proportion of the basal subtype (43%) in the younger cytokine-positive group (Figure 3B). We evaluated the overall survival in the cohort for patients >55 and <55 years of age and with overexpression or underexpression (defined as a z-score < −1) of any of the four cytokines, and found that overexpression of the cytokines preferentially influence the survival of patients under 55 years old (Figure S3). Most notably, we found that overexpression of GM-CSF (HR = 1.67, 95% CI = 1.01–2.77, *p* = 0.042) and RANTES (HR = 1.8, 95% CI = 1.01–3.21, *p* = 0.043) correlated with decreased overall survival in the <55 years group (Figure 3C). Survival curves for MCP-1 were similar to those of GM-CSF and RANTES (though the difference was not significant; *p* = 0.32). Conversely, the survival curves were inverted with overexpression of G-CSF, though the difference was not significant (HR = 0.87, 95% CI = 0.51–1.49, *p* = 0.62; Figure 3C).

We also performed a principal component analysis (PCA) and K-mean cluster analysis to investigate the distribution of the four cytokines in each BRCA subtype in patients under 55 years of age, using expression data for each cytokine and subtype data as input to construct the PCA. We observed that PC1, which explained the greatest variation of data (38.12%), separated the samples in two groups, one harboring the aggressive basal and Her2-enriched BRCA subtypes, and the other harboring the non-aggressive luminal and normal subtypes. PC1 also showed negative loading for GM-CSF, MCP-1, and RANTES, and positive loading for G-CSF. The first three cytokines clustered with the aggressive BRCA subtypes, while G-CSF clustered with the non-aggressive subtypes, in agreement with the overall survival analysis (Figure 3D). Indeed, a survival analysis performed on samples overexpressing only the three cytokines GM-CSF, RANTES, and MCP-1 correlated better with decreased survival (*p* = 0.05 and HR of 1.33, 95% CI = 0.99–1.78; Figure 3E), than an analysis based on the expression of all four cytokines (*p* = 0.092 and HR of 1.26, 95% CI = 0.96–1.67; Figure S3). Overall, these analyses suggest that the cytokines GM-CSF, RANTES, and MCP-1 are overexpressed in BRCA subtypes with an unfavorable outcome while G-CSF is not.

### 2.4. An Extended Gene Signature Associated with M1-Like Macrophages Is Enriched in Claudin-Low Breast Tumors

**Figure 4:**
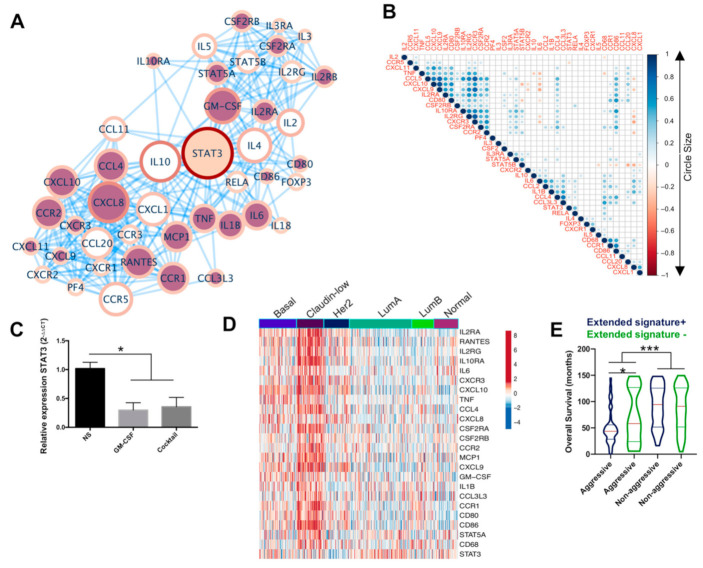
An extended gene expression signature associated with differentiation and polarization of M1-like macrophages is enriched in claudin-low tumors. (**A**) A protein–protein interaction network was generated using GM-CSF, MCP1, and RANTES as input. The size of the node and the color gradient at the border represent closeness centrality and betweenness centrality, respectively; the nodes filled with red or cream represent the genes with a significant positive and negative expression correlation, respectively; white-filled nodes were not significant. (**B**) Matrix of expression correlation of the interaction network components in the METABRIC breast cancer database, genes with a significant positive or negative correlation within samples were represented in blue and red, respectively, and the circle size and the color intensity are proportional to the Pearson’s correlation coefficient. Empty boxes are non-significant correlations (*p*-value > 0.01). (**C**) Relative expression of STAT3 in M1-like macrophages measured by RT-PCR. (**D**) Gene expression of the M1-like macrophage extended signature in patients younger than 55 years old. Heatmap was clustered by gene expression correlation and BRCA subtype. (**E**) Survival of patients divided by aggressive (basal, claudin-low, and Her2-enriched) or non-aggressive (luminal A, luminal B, and normal) subtypes and by the positive (samples with one SD above the median value of NES) and negative (samples with one SD below the median value of NES) occurrence of the extended signature. NES: Normalized Enrichment Score, SD: standard deviation. Data represent the mean ± SEM (standard error of the mean); non-parametric Kruskal–Wallis and Dunnett post-hoc tests, * *p* < 0.05 and *** *p* < 0.001.

To better understand the role that GM-CSF, RANTES, and MCP1 play in BRCA, we generated a protein–protein interaction network (PPI), using String [47] and Cytoscape [48] software to evaluate the signaling pathways and biological processes in which these cytokines participate. The PPI showed 42 nodes, with loss of STAT3 as the most central node in the network determined by closeness and betweenness centrality (Figure 4A). We analyzed a Pearson correlation matrix to determine positive and negative expression correlations within the network genes in the BRCA samples of METABRIC. This analysis showed that 24 genes had a positive correlation and STAT3 had a negative correlation (R = −0.38, *p* = 0.005; Figure 4B). Gene ontology and pathway analyses showed that the network of 24 genes was associated with processes including monocyte chemotaxis, differentiation, and polarization of M1 macrophages (Figure S4A) while STAT3 was associated with M2 macrophages, in agreement with loss of this gene in the interaction network [49,50,51]. Loss of STAT3 in the M1-like macrophages was confirmed by RT-PCR (Figure 4C).

We proposed that the 24 genes with a positive correlation plus the loss of STAT3 could constitute an expression signature that favors the differentiation of peripheral monocytes arriving in the TME into M1-like macrophages. We assessed the expression of these 24 genes in BRCA subtypes as an M1-like extended signature, also hypothesizing that samples enriched in this signature should mark intrinsically aggressive subtypes. We observed that this extended gene signature is highly represented in the claudin-low subtype, one of the BRCA subtypes with the worst prognoses [52,53], which in our previous analyses was part of the basal samples (Figure 4D). Claudin-low samples represent 11% of the BRCA samples in patients younger than 55 years, and of these, 85% are positive for the extended signature (Figure S4B). To determine positivity for the extended signature, we performed a single-sample gene set enrichment analysis (ssGSEA) (described below), considering positive samples as those one standard deviation (SD) above the median value of the normalized enrichment score (NES) and negative samples as those one SD below the median NES value. Although the claudin-low patients positive for the extended signature exhibited poor overall survival (median of 45.6 months vs. 101.2 months for those without the extended signature; Figure S4B and Table S2), due to the low number of claudin-low samples negative for the signature (*n* = 4), we were not able to perform a statistically robust survival analysis. However, when we compared the median overall survival of all patients divided into aggressive (basal, claudin-low and Her2-enriched) and non-aggressive (lumA, lumB, and normal) subtypes, we observed that patients with the extended signature and an aggressive subtype have significantly lower survival (median of 43.5 months) than those who have an aggressive subtype without the signature (median of 58.3 months), or who present a non-aggressive subtype with or without the signature (Figure 4E).

We also analyzed the overall survival of the full cohort of METABRIC BRCA patients without age restrictions, based on the presence of the extended signature (Figure S4C). We observed a similar pattern in the full cohort compared to the <55 age group, with patients positive for the extended signature showing a tendency towards reduced overall survival. The percentage of claudin-low samples was similar in both groups, but claudin-low samples positive for the extended signature were enriched in the younger group (85% vs. 70%). The median overall survival in the full cohort was 78.93 and 101.22 months for claudin-low with and claudin-low without the signature, respectively (Figure S4C and Table S2). We could not perform a statistically robust analysis here either due to the reduced number of samples. These results suggest that cytokines GM-CSF, MCP-1, and RANTES along with the extended signature based on their interacting proteins are highly represented in aggressive BRCA, particularly in claudin-low tumors, and correlate with reduced overall survival. The enrichment of this signature with monocyte chemotaxis, macrophage differentiation, and polarization into the M1 subtype also agrees with our initial analysis of the participation of GM-CSF, MCP-1, and RANTES in the formation of M1-like macrophages.

### 2.5. The M1-Like Macrophage Extended Signature Concur with Th1 and Immunosuppressive Signatures in Claudin-Low Tumors Denoting Poor Prognosis

**Figure 5:**
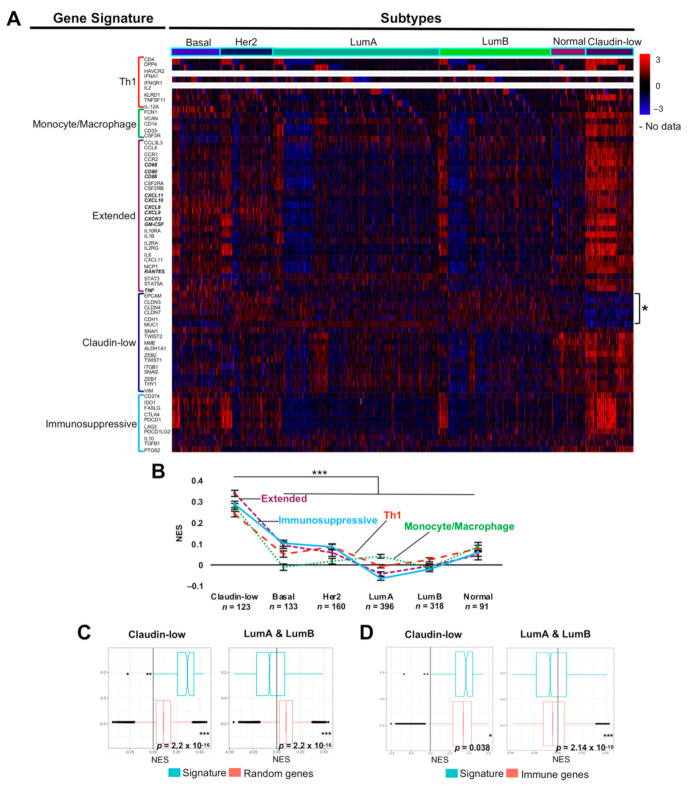
The M1-like macrophage extended gene signature co-occurs with a Th1 and immunosuppressive signatures in claudin-low breast cancers. (**A**) Heatmap of a supervised clustering showing convergence in claudin-low samples of a Th1 (red bracket), monocyte/macrophage (green bracket), claudin-low (blue bracket; the lost of claudins and other epithelial markers is indicated by *), immunosuppressive (aquamarine bracket), and M1-like macrophage extended (purple bracket) gene signatures, the full cohort of METABRIC BRCA samples was used (*n* = 1221). Genes in italic and bold are shared between the Th1, monocyte/macrophage and the extended signatures, but were only represented in the M1-like microphage extended signature. (**B**) Single-sample gene set enrichment analysis of Th1, monocyte/macrophages, immunosuppressive, and M1-like extended signatures for each one of the BRCA subtypes, colors for each signature are as in (**A**). NES values for all signatures in each BRCA subtype were compared with a non-parametric Kruskal–Wallis and Dunnett post-hoc test. Comparison of NES values by permutation analysis of the extended signature in claudin-low and luminal subtypes using randomly sampled genes (**C**), or immune-related genes (**D**), using the Mann–Whitney-Wilcoxon test. NES: Normalized Enrichment Score. * *p* < 0.05 and *** *p* < 0.001.

Macrophages referred to as M1 and M2 were originally discovered as being associated with Th1 and Th2 immune responses, respectively [54]. Therefore, we analyzed the full cohort of 1221 BRCA samples to determine if there was a correlation between Th1 responses and the extended M1-like signature, using the Th1 gene signature proposed by Jerby-Arnon et al. [55]. We noticed that this Th1 signature includes RANTES receptors CCR1 and CCR5. We also tested our extended signature against a proinflammatory monocyte/macrophage signature proposed by single-cell RNA sequencing of the immune cell infiltrate in melanoma [56], and against a shortened signature that identifies the claudin-low BRCA subtype, based on loss of claudins 3, 4, and 7 and other adhesion genes (marked with an asterisk in Figure 5A) and gain of mesenchymal genes [57]. A correlation between high immune cell infiltration and a high immunosuppressive score has been reported for other carcinomas to explain tumor immune escape in spite of an immune cell-rich TME; thus, we also included an immunosuppressive signature in this analysis [58] (see Table S3 for the genes included in each gene signature). A heatmap generated with the METABRIC BRCA samples showed the co-occurrence of all signatures with the claudin-low subtype (Figure 5A). An ssGSEA confirmed that the Th1, monocyte/macrophage, immunosuppressive, and extended M1-like macrophage signatures were significantly enriched in the claudin-low subtype compared with the other subtypes (Figure 5B), with the M1-like extended signature exhibiting the highest NES.

We verified that the M1-like signature was not explained by random associations or by the enrichment of the immune infiltrate by comparing the signature against 1000 permutations of random and immune-related gene sets. In the former analysis, we confirmed the significant enrichment of the M1-like extended signature with claudin-low samples (*p* = 2.2 × 10^−16^), while the luminal samples exhibited an exclusion of the signature (*p* = 2.2 × 10^−16^) (Figure 5C). The same conclusion was reached by comparison with immune-related genes, supporting the idea that the extended M1-like macrophage signature is enriched in claudin-low BRCA samples (*p* = 0.038) and excluded in luminal samples (*p* = 2.14 × 10^−10^; Figure 5D). Interestingly, the comparison with immune-related genes showed basal BRCA samples to also be enriched with the M1-like signature (*p* = 0.018; Figure S5). This may be related to the unfavorable overall survival that we also observed in patients with basal BRCA who were positive for the extended M1-like signature (Figure 4E).

**Figure 6:**
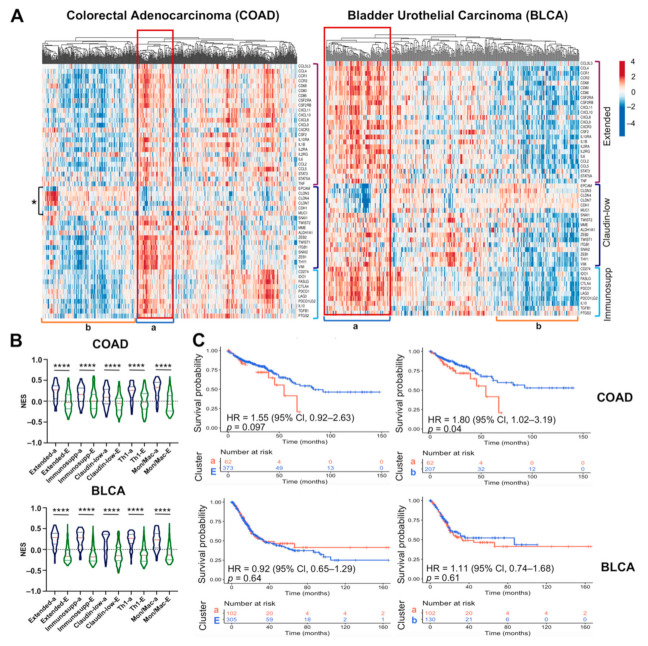
A claudin-low subtype is present in colorectal and bladder carcinomas. (**A**) Heatmap and (**B**) NES of the M1-like macrophage extended, immunosuppressive, claudin-low (the lost of claudins and other epithelial markers is indicated by *), Th1, and monocyte/macrophage gene signatures in patients with COAD (*n* = 435) and BLCA (*n* = 407). The Mann–Whitney-Wilcoxon test was used to compare NES between the cluster positive to all signatures (a) vs. remaining samples (E). (**C**) Kaplan–Meier survival curves were performed with COAD and BLCA samples. We made two comparisons, the cluster positive (one SD above the median value of NES) to all signatures (a) vs. remaining samples (E) (left plot) or vs. samples with one SD above the median value of NES to all signatures (b) (right plot). *p*-values were calculated using the Log-Rank test and the hazard ratios (HRs) were determined with 95% confidence intervals (95% CI). Immunosupp: Immunosuppressive, Mon/Mac: Monocyte/Macrophage, NES: Normalized Enrichment Score, SD: standard deviation. **** *p* < 0.0001.

A claudin-low subtype, also known as the mesenchymal subtype [59], has also been reported in COAD and BLCA [58], which are also molecularly characterized by the loss of expression of *CLDN3, CLDN4, CLDN7* (claudins 3, 4, and 7), *CDH1* (E-cadherin), *MUC1*, and *EPCAM*, and gain of *VIM* (vimentin) and several other mesenchymal markers [58,59,60]. We analyzed whether the M1-like macrophage extended signature also correlates with the COAD and BLCA claudin-low/mesenchymal subtype, finding that samples with the extended signature were also related to the claudin-low, immunosuppressive, Th1, and monocyte/macrophage signatures (Figure 6A,B). In addition, we found that COAD patients positive for the mesenchymal/inflammatory/immunosuppressive signatures had worse overall survival than patients negative for these signatures (*p* = 0.04 and HR of 1.8, 95% CI = 1.02–3.19). Surprisingly, the BLCA analysis did not show a significantly decreased survival in patients harboring the signatures (Figure 6C). Altogether, these data argue that the extended M1-like signature is enriched in claudin-low tumors, and that in BRCA and COAD, this signature may be involved in poor clinical outcome.

## 3. Discussion

We have previously reported that triple-negative BRCA with increased levels of GM-CSF, G-CSF, MCP-1, and RANTES promotes the formation of macrophages with protumor activity [3,35,37]. In this study, by stimulating PBM with these cytokines at the concentrations found in supernatants of the BRCA cells, we replicated our earlier data. This treatment generated macrophages that induced luminal BRCA cells to underexpress E-cadherin and overexpress SOX-2 and OCT-4, turning them into invasive cells. In previous studies, we also compared a battery of macrophage differentiation stimuli and observed the formation of macrophages that co-expressed M1 and M2 markers, particularly of markers commonly used to set them apart (e.g., CD163, CD36, arginase, iNOS, IL-10, TNFα, and VEGF) [35]. In our experimental setting, the best markers that discriminate between M1/proinflammatory and M2/anti-inflammatory stimuli were IL-8 (for M1) and EGF (for M2) [3,35]. Macrophages induced by the four recombinant cytokines seem to be close to the M1-like macrophages that we have previously observed (IL-8^pos^ and EGF^neg^).

The study of TAMs has long been framed by the assessment of these two populations of macrophages. High infiltration of M2 macrophages [61] or a low M1/M2 ratio have been found to correlate with poor prognosis in patients with breast [38], ovary [39,40], stomach [41], and cervical cancers [42]. In medulloblastoma, the opposite has been observed, with a high M1/M2 ratio correlating with poor prognosis [62]. Thus, in this dichotomic classification, M1 and M2 macrophages are more commonly associated with good and bad prognosis, respectively. More recent studies have used single-cell technologies to characterize TAM populations in greater depth. These studies support the existence of more than two different types of TAMs as defined by transcriptional and proteomic signatures. Interestingly, in BRCA, TAMs could not be assigned to M1 or M2 subtypes, with single TAMs co-expressing broadly used markers of both [63,64,65]. Furthermore, many markers that were considered unique to macrophages are now known to be expressed by multiple immune cell lineages, e.g., CD68/CD14, CD11b, CD11c, HLA-DR, and IL-4RA [66]. This has perhaps contributed to inaccurate assignments of pro- and antitumoral roles. Unlike terminally differentiated lymphoid populations, which are highly modified epigenetically, macrophages exist in a continuum of different polarization stages and remain plastic against different activating stimuli [67,68,69]. M1 classical and M2 alternative states are most likely found only at the poles of such a continuum. In this scenario, macrophages closer to the M1 or M2 poles may be relevant in some pathological and/or physiological conditions. In cancer, in which multiple inflammatory and anti-inflammatory stimuli coexist, well-defined states of polarization may be more difficult to achieve, and tumors probably nurture a mix of M1 and M2 activities. Since M2-to-M1 reprogramming has been proposed as an anticancer therapy, it is important to establish whether exclusive M1 and M2 TAMs exist, and whether they fulfill fixed antitumoral or protumoral functions.

Although G-CSF was among the prominent cytokines secreted by the triple-negative BRCA in our previous studies, this cytokine was not associated with the most aggressive intrinsic BRCA subtypes and did not seem to influence patient survival. Conversely, multiple lines of evidence support protumoral functions for GM-CSF, MCP-1, and RANTES. RANTES has been linked to progression of triple-negative BRCA and to the relapse of BRCA patients, along with MCP-1 [70,71]. MCP-1 is a potent growth factor of tumor cells and promotes invasion and metastasis, particularly in triple-negative BRCA [72,73]. Co-expression of MCP-1 and RANTES inhibits cytotoxic T cells in advanced BRCA [74]. GM-CSF activates and expands myeloid-derived suppressor cells (MDSCs), also contributing to an immunosuppressive TME [75]. We related GM-CSF, MCP-1, and RANTES with an extended molecular signature that participates in monocyte chemotaxis, differentiation, and polarization into macrophages closer to the M1 phenotype. For instance, the transcription factor STAT5, which is downstream of GM-CSF receptor signaling, has been shown to drive M1 macrophage polarization [76,77]. On the other hand, the three cytokines are linked to underexpression of STAT3, a transcription factor related to M2 macrophage polarization [49,50,51]. Components of the extended signature (CD80, CD86, IL-1β, IL-2, IL-6, and TNFα) are also linked to M1 macrophages, either as markers or as signaling intermediaries. GM-CSF, MCP-1, and RANTES together with CCL3L3 and CCL4 (MIP-1β) chemoattract and/or activate PBM at the tumor site (see Figure S3A,B, and [78]).

To a large extent, the tumor antagonist/agonist activities of macrophages have been explained by a core of proinflammatory properties in M1 TAMs, and anti-inflammatory properties in M2 TAMs [79]. However, this is inconsistent with the substantial evidence supporting inflammation as a hallmark of cancer [80], and the maintenance of both types of responses in the TME through all stages of tumor initiation and progression [81]. Inflammation facilitates cellular mutagenesis through production of reactive oxygen and nitrogen species. These are most likely a critical driver for carcinogenesis but may also facilitate tumor evolution by promoting intratumoral heterogeneity and the acquisition of novel aggressive functions [81,82]. Proinflammatory cytokines and their downstream signaling pathways and transcription factors also contribute to cancer progression [6,81]. The extended M1-like signature is certainly more related to a proinflammatory than an anti-inflammatory TME.

M1 and M2 macrophages were originally described as metabolically distinct subclasses based on their ability to process arginase into nitric oxide or ornithine, respectively [54]. Mills et al. labeled them as M1 and M2 to mirror the Th1 and Th2 T cell nomenclature, since M1 macrophages were more consistently derived from mice with preferential Th1 responses, and vice versa for M2 macrophages [54]. Consistent with this, we observed that the Th1 and the extended M1-like macrophage signatures were admixed in claudin-low triple-negative BRCA. Claudins are small transmembrane proteins that are the backbone of tight junctions, protein complexes that seal and compartmentalize epithelial cells by forming tissue and organ barriers that impede free diffusion of solutes and water. Tight junctions hold together the epithelial cell barriers, a characteristic of terminally differentiated cells. Hence, claudin-low tumors are poorly differentiated. The loss of claudin expression (mainly claudins 3, 4, and 7) correlates with EMT (underexpression of E-cadherin and overexpression of mesenchymal markers) and stemness (SNAI1, SNAI2, TWIST1, TWIST2, ZEB1, and ZEB2; see Table S3) [52,57]. Thus, claudin-low BRCA resembles breast progenitor cells [52,83]. There is evidence suggesting that claudin-low BRCA originates by direct transformation of normal stem/progenitor cells but also from more differentiated cells influenced to reprogram/de-differentiate by driver mutations or environmental conditions [43,84,85,86,87]. The capacity of cytokine-induced M1-like macrophages to alter expression of EMT/stem markers supports the latter. It is also relevant that we have previously generated M1-like macrophages with stimuli derived from MDA-MB-231 and Hs578T cells [3]. These two BRCA cell lines were previously identified as claudin-low by gene expression signatures, such as low expression of epithelial cell adhesion genes and luminal markers, and high expression of stem markers [52].

The claudin-low BRCA subtype has the highest density of immune cell infiltrate [57,88,89]. Th1 responses include cytokines that nurture the activity of cytotoxic T cells, and as such, are more often referred to as antitumoral. Th1 response genes include those for IFNγ, IL-2, IL-12, and CD94 (KLRD1) receptors, which are important drivers of T cell and NK cell cytotoxicity. Many tumor mechanisms are known to circumvent immune cytotoxic responses, such as a decrease in human leukocyte antigens (HLA) and IFNγ expression; decreased neoantigen load in genetically stable tumors; an immunosuppressive TME rich in TGFβ/IL-10, IDO, or Treg/MDSC populations; decreased expression of T cell receptor coactivators; and increased expression of checkpoint inhibitors [11,90,91]. Jerby-Arnon et al. found that absence of the Th1 signature explains the immunotherapy resistance of melanomas [55]. Claudin-low tumors are also characterized by being genomically stable, a feature also attributed to their progenitor origin [83,92]. Remarkably, we also found that in claudin-low tumors, the Th1 and extended M1-like macrophage signatures co-exist with an immunosuppressive signature that includes TGFβ/IL-10/IDO molecules and multiple checkpoint inhibitors (see Table S3). Lack of somatic hypermutation together with the immunosuppressive signature may allow the co-existence of tumor cells with the abundant immune infiltrate.

The BRCA claudin-low immune infiltrate is still poorly characterized. Bareche et al. found a correlation between BRCA with mesenchymal and mesenchymal/stem-like features and a TME enriched in innate immune cells [93], but they did not explore the diversity of the populations present, or characterize the macrophages in detail. Claudin-low tumors were originally described in BRCA but then also found in BLCA, in which they are also characterized by a mesenchymal and stem-like phenotype and a high immune infiltrate [58]. Three intrinsic subtypes have been proposed for BLCA: basal, luminal, and claudin-low, which are transcriptionally similar to their BRCA counterparts [94]. The analysis of the BLCA TME showed that each subtype has a specific microenvironment, with claudin-low tumors enriched with an immune cell infiltrate in which macrophages and Th1 responses are highly represented [58], similar to what we observed in BRCA. Although unrecognized as claudin-low, there is a COAD subtype with mesenchymal features in which the TME has been characterized in great depth [95]. Similar to claudin-low BRCA and BLCA, mesenchymal COAD (also called CMS4) is characterized by having a large immune infiltrate rich in macrophages, monocytes, Tregs, Th17 cells, and MDSCs [96,97]. Differential gene expression analysis of 1388 COAD samples also showed a correlation between CMS4 and enrichment of cells of monocytic origin as well as lymphocytes, and between inflammation and immunosuppression [98]. One COAD study observed that alterations in the TME identified patients that would develop metastasis, and macrophages were one of the cell populations better representing changes in the TME [99]. CMS4 also has the worst patient survival of all COAD subtypes [96,97].

To date, the mechanisms linking a tumor mesenchymal/stem-rich profile and a high density of immune cell infiltrates are unknown. Even so, it is interesting to note that IL-8, TNFα, IL-1β, and IL-6 proinflammatory cytokines, known to be secreted by M1 macrophages, activate signaling pathways related to EMT, such as the JAK-STAT, NFκB, mitogen activated kinases (MAPKs), and PI3K/AKT/β-catenin [43,100,101,102]. Cells experimentally induced to undergo EMT also upregulate the expression of stemness factors OCT4, SOX2, NANOG, and CD44 [43,103]. We have previously shown that the M1-like macrophages induced by triple-negative BRCA express high levels of IL-8, TNFα, and IL-1β [3,35,37]. In addition, activation of JAK-STAT, NFκB, and MAPKs were terms linked to the extended M1-like macrophage signature in our gene ontology and KEGG analyses. STAT3 seems to be an essential intermediate when EMT is induced through IL-8/IL-6 [104,105,106,107]. Though this transcription factor was a central molecule in the extended M1-like signature, it was downregulated in the interaction network. STAT3 has also been related to M2 macrophage polarization [49,50,51], and in support of this we observed STAT3 downregulation in the cytokine-induced M1-like macrophages. IL-8, IL-1β, MCP-1, RANTES, TNFα, and IL-6 also promote tumor cell migration and invasion [108,109]. We have also documented that the induced M1-like macrophages secrete high levels of metalloproteinases and COX-2 [3,37]. The former degrade the extracellular matrix, facilitating invasion, and the latter is highly expressed in human and murine claudin-low tumors [110].

Other interesting components of the extended M1-like signature are the chemokine receptor CXCR3 and its ligands CXCL9, CXCL10, and CXCL11. Whereas the chemokines are expressed by myeloid cells in response to pathogen-associated molecular pattern (PAMP) stimuli, CXCR3 is expressed in T cells and NK cells in response to interferons [111]. Thus, the CXCR3 axis regulates the recruitment and activation of NK cells, CD8 T cells, and CD4 Th1 cells, and multiple lines of evidence support the activity of this axis in tumor control [112,113,114,115,116]. More recently, an opposite activity promoting tumor growth has also been documented [111,117]. In triple-negative BRCA, CXCL10 and CXCR3 were shown to contribute to tumor invasiveness and metastasis in spite of a high density of T cell infiltrate [118]. CXCL10 and CXCL11 increase the number of CXCR3-expressing cancer stem cells in BRCA cell lines [119]. CXCR3 is highly expressed in high-grade BRCA [120] and its expression level is an indicator of poor prognosis [121]. A polymorphism that increases CXCL10 levels in circulation is over-represented in patients with advanced BRCA [122]. Other studies support the idea that increased levels of CXCL10 mark more advanced BRCA [123], and that overall, the CXCR3 axis increases BRCA metastasis. The use of antagonists of this axis has been proposed to inhibit lung metastasis [124,125]. Certainly, increased tumor immune infiltration provides better targets for immunotherapy, and our findings open a window to better characterization of the claudin-low TME. There could be an opportunity to develop immunotherapy strategies that improve the prognosis of patients, particularly considering that most BRCA has been unresponsive to immunotherapy targeting PD-1 and CTLA4 checkpoints [12,13], and only PD-L1-positive recurrent unresectable or metastatic triple-negative BRCA has been approved for treatmeant with pembrolizumab (anti-PD-1) or atezolizumab (anti-PD-L1).

## 4. Materials and Methods

### 4.1. Cell Culture and Generation of PBM

All human BRCA cell lines were obtained from the American Type Culture Collection (ATCC), and their short tandem repeat profiles are routinely tested to verify their authenticity. The BRCA cell lines used are MCF7, T47D, and MDA-MB-231. Each cell line has been classified according to their aggressive features, and those considered aggressive are positive to the stem marker CD44, positive to the mesenchymal marker vimentin while negative to the epithelial marker E-cadherin, and exhibit invasive capacity in Matrigel-based transwell assays. Thus, MCF7 and T47D cells are classified as non-aggressive BRCA, and MDA-MB-231 as aggressive. MCF7 cells were cultured in DMEM high in glucose (4.5 g/L), T47D cells in RPMI 1640, and MDA-MB-231 in DMEM/F12 medium. PBM studies were carried out with cells derived from three different healthy volunteers. PBMs were isolated by differential separation using Histopaque 1077 (Sigma-Aldrich, St. Louis, MO, USA) and the monocyte-enriched fraction was obtained by negative selection using the Pan-Monocyte Isolation Kit (Miltenyi Biotec, Bergisch Gladbach, Germany). Monocytes were cultured in DMEM/F12. U937 monocytes were cultured in RMPI 1640 and used for migration assays. All culture media were supplemented with penicillin 100 U/mL, streptomycin 100 µg/mL, fungizone 0.25 µg/mL, and 10% fetal bovine serum (FBS), with the exception of monocytes that were cultured with 6% FBS during the differentiation process. Culture media and supplements were obtained from Gibco BRL Life Technologies (Grand Island, NY, USA). All cells were cultured at 37 °C in 5% CO_2_.

MCF7 and T47D cells were used to authenticate the capacity of polarized macrophages to promote aggressive features into non-aggressive BRCA, while MDA-MB-231 cells were used as control of induced aggressive features. For this, 5 × 10^4^ non-aggressive cells were seeded on a coverslip and kept for 24 h, after which culture media were discarded, cells were rinsed with PBS 1X, and cultured with conditioned media from cytokine-polarized macrophages (as described below) for 48 h. The following aggressive features were evaluated on induced non-aggressive BRCA cells: invasion capacity by Matrigel-based transwell assays, expression of E-cadherin and vimentin EMT markers, and expression of OCT-4 and SOX-2 stemness markers by immunofluorescence (described below).

### 4.2. Macrophage Differentiation and Polarization

First, 2 × 10^5^ monocytes/well were plated in a 24-well culture plate. Monocytes were stimulated with the four cytokines individually or with a cocktail of all of them for 6 days. The concentrations tested for each cytokine were those previously determined in supernatants of aggressive BRCA cells, both commercial and primary cells isolated from BRCA patients: 5 ng/mL GM-CSF, 5 ng/mL G-CSF, 10 ng/mL MCP1, and 1 ng/mL RANTES (all cytokines were from Miltenyi Biotec, Bergisch Gladbach, Germany) [3]. To obtain the conditioned media of induced macrophages, first, the culture media with the cytokines was discarded and macrophages were rinsed with PBS 1X, and cultured in serum-free DMEM/F12 medium for an additional 48 h. Then conditioned media were harvested, centrifuged at 1500× *g* rpm/5 min, filtered over a 0.22 μm membrane to remove cells, and debris, aliquoted, and stored at −20 °C until use. To evaluate whether we differentiated PBM into macrophages, we evaluated positivity to CD14 and CD68 by flow cytometry, and to ascertain whether those macrophages were polarized into M1- or M2-like subtypes, we measured IL-8 and EGF expression, respectively (see below). As comparison controls, macrophages M1 and M2 were generated using standard polarization conditions, as previously documented [35]. Briefly, 2 × 10^5^ monocytes/well were plated in a 24-well culture plate and stimulated with 100 ng/mL of GM-CSF (Miltenyi Biotec, Bergisch Gladbach, Germany) for 6 days, followed by 100 ng/mL of LPS (Sigma-Aldrich, St. Louis, MO, USA) and 25 ng/mL of IFN-γ (R&D systems, Minneapolis, MN, USA) for 48 h (condition M1), or monocytes were stimulated with 100 ng/mL of M-CSF (PeproTech Inc., Rocky Hill, NJ, USA) for 6 days, followed by 25 ng/mL of IL-4 (Sigma-Aldrich, St. Louis, MO, USA) and 25 ng/mL of IL-13 (Sigma-Aldrich, St. Louis, MO, USA) for 48 h (condition M2).

### 4.3. Migration Assay

Since G-CSF, MCP-1, and RANTES are chemokines known to attract myeloid cells, to validate the activity of the recombinant cytokines we performed a migration assay with the U937 monocytic cell line. We used the three cytokines as chemotactic agents at the concentrations that we used in the cocktail, and we additionally used 100 ng/mL of each cytokine that is the concentration traditionally used in other studies [3]. We placed 1.5 × 10^5^ U937 cells in 200 μL of serum-free RPMI medium in transwell inserts with 3-μm pores, and in the lower chamber, we placed 800 μL of serum-free medium supplemented with the corresponding cytokine. Cell migration was allowed to progress for 24 h at 37 °C in a humidified 5% CO2 environment. Migratory cells were counted at 24 h using a microscope Motic AE31, and images were acquired with a digital camera (Moticam 5.0 MP). The cell count was performed in FIJI ImageJ software.

### 4.4. Quantitative Determination of IL-8

The production of IL-8 was determined in the conditioned media of the induced-macrophages using the Human IL-8 ELISA Kit (BD Biosciences, San Diego, CA, USA), and following the manufacturer’s instructions.

### 4.5. Flow Cytometry

For extracellular staining of CD14, CD86, CD163, and CD206, 2 × 10^5^ cells were used per assay and the unspecific binding was blocked with 50% FBS in PBS for 15 min, then cells were stained with mouse monoclonal anti-CD14-FITC antibody (1:50, BD Biosciences, San Diego, CA, USA), mouse monoclonal anti-CD86-PE (R&D systems, Minneapolis, MN, USA), mouse monoclonal anti-CD163-PerCP (R&D systems, Minneapolis, MN, USA), and mouse monoclonal anti-CD206-FITC (BD Biosciences, San Diego, CA, USA) for 30 min. Finally, cells were incubated with 7AAD (7-amino-actinomycin, BD Biosciences, San Diego, CA, USA) to exclude dead cells. An intracellular staining protocol was used for the CD68 marker as follows: 2 × 10^5^ cells were incubated for 4 h with brefeldin A (Biolegend, San Diego, CA, USA), after which, the cells were harvested with Accutase protease (Sigma-Aldrich, St. Louis, MO, USA). Unspecific binding was blocked with 50% FBS in PBS for 15 min, and then, cells were fixed and permeabilized for 30 min with the fixation/permeabilization solution kit (BD Biosciences, San Diego, CA, USA), cells were rinsed with Perm/Wash buffer 1X (BD Biosciences, San Diego, CA, USA), blocked again with 50% FBS (diluted in perm/wash buffer 1X) for 15 min, and incubated with mouse monoclonal anti-CD68-FITC antibody (1:100, BD Biosciences, San Diego, CA, USA) for 30 min. The samples were acquired with the Guava Easycyte 8 cytometer (Merck Millipore, Darmstadt, Germany) and analyzed with the FlowJo V.10.0.7 software.

### 4.6. Immunofluorescence Assay

After non-aggressive BRCA cells were stimulated for 48 h with the M1-like macrophages conditioned media, they were fixed with paraformaldehyde 4% and permeabilized with 0.5% TritonX-100-SDS at room temperature. Three percent of bovine serum albumin (BSA) was used to block unspecific binding. The proteins of interest were stained with either of the following primary antibodies: mouse monoclonal anti E-Cadherin (1:100, BD Biosciences, San Diego, CA, USA), rabbit monoclonal anti-vimentin-Alexa Fluor-594 (1:1000, Abcam, Cambridge, MA, USA), rabbit monoclonal anti-OCT4 (1:100, Abcam, Cambridge, MA, USA), and mouse monoclonal anti-SOX2 (1:100, Abcam, Cambridge, MA, USA) for 1 h or overnight (stemness factors). We evaluated EGF expression to assess whether the induced macrophages were M1- or M2-like. For this, macrophages were treated as non-aggressive BRCA cells and then stained with mouse monoclonal anti-EGF-PE (1:100, Santa Cruz Biotechnology, Santa Cruz, CA, USA) for 1 h. Cells were then rinsed with PBS 1X, and stained with secondary antibodies: donkey anti-mouse-Alexa Fluor 647 (1:200, Jackson ImmunoResearch Laboratories, Inc., West Grove, PA, USA), or donkey anti-rabbit-FITC (1:200, Jackson ImmunoResearch Laboratories, Inc., West Grove, PA, USA). DAPI (0.4 µg/mL, Thermo Fisher Scientific, Eugene, OR, USA) for 1 h was used to counterstain nuclei. The preparations were mounted using Vectashield to preserve fluorescence. Images were acquired on a Nikon Ti Eclipse inverted confocal microscope equipped with an A1 imaging system, using the software NIS Elements v.5.0. Fluorescent and optical images were recorded using a 20X (Numerical aperture = 0.8) objective lens and Nyquist zoom. Fluorescence was excited using the 405 nm (DAPI), 488 nm (FITC), 561 nm (AF594), and 633 nm (AF647) laser lines. Emissions were read in the 425–475 nm (DAPI), 500–550 nm (FITC), and 663–740 nm (AF647) ranges. The integrated density was calculated using a default threshold value in all images, and the analysis was performed in the FIJI ImageJ software. The integrated density was calculated in three fields (20X) per condition; all experiments were performed by triplicate.

### 4.7. Invasion Assay

Cytokine-polarized macrophages were rinsed with PBS 1X, resuspended in 800 µL of serum-free DMEM/F12 medium, and placed in the bottom part of an 8-µM pore transwell plate containing Matrigel (1:4, BD Biosciences, San Diego, CA, USA). Then, 5 × 10^5^ BRCA (non-invasive) cells in 200 µL of serum-free DMEM/F12 medium were placed in the top part of the transwell and the assay was left to proceed for 48 h. The invading cells were fixed with 4% paraformaldehyde for 20 min and stained with violet crystal (1:250) for 30 min. The number of invading non-aggressive BRCA cells was determined by counting three random fields per condition; all experiments were performed by triplicate.

### 4.8. Quantitative RT-PCR

Total RNA was isolated from 1 × 10^6^ cytokine-polarized macrophages using the RNeasy Mini Kit (Qiagen, Germantown, MD, USA), and quantified in a NanoDrop OneC spectrophotometer (Thermo Fisher Scientific, Madison, WI, USA). Then, 1 µg of isolated RNA was used to synthesize cDNA for each sample using the RT2 First Strand Kit (Qiagen, Hilden, NRW, Germany) and following the manufacturer’s instructions. The following primers were used: for STAT3 amplification, 5′-GCTTTTGTCAGCGATGGAGT-3′ (forward), 5′-ATTTGTTGACGGGTCTGAAGTT-3′ (reverse) [126,127], and for the GAPDH housekeeping control, 5′-CTTCACCACCATGGAGAAGGC-3′ (forward), 5′-GGCATGGACTGTGGTCATGAG-3′ (reverse) [128]. The PCR reaction mixture was performed in a final volume of 12.5 µL, which contained 2X SYBR^®^ Green PCR Master Mix (Applied Biosystems, Carlsbad, CA, USA), 250 nM of each primer, and 25 ng of cDNA. The following thermal cycling was employed, an initial step at 95 °C for 10 min; followed by 50 denaturation cycles at 95 °C for 20 s, annealing at 59 °C for 20 s and extension at 72 °C for 20 s. The amplification was performed in a Rotor-Gene Q 5 PLEX HRM (Qiagen, Hilden, NRW, Germany) and was analyzed in the Rotor-Gene 114 Q Software 2.3 (Qiagen, Hilden, NRW, Germany). The 2^−ΔΔCt^ method was used to determine relative expression.

### 4.9. METABRIC and TCGA Data Analysis

The clinical significance of the four cytokines able to polarize macrophages into protumoral subtypes was evaluated using public database from TCGA and METABRIC. The BRCA database include normalized expression data (Illumina HT 12 arrays), clinical data related to the patient’s overall survival, and the PAM50 intrinsic classification of the tumor based on expression of hormone receptors, Her2, and claudins. cBioPortal for Cancer Genomics (www.cBioPortal.org, retrieved August 2020) was used to access to METABRIC and TCGA data. All analyses were performed with the normalized z-score available on the website; a z-score threshold ±1 for RNAseq (TCGA) and for microarrays (METABRIC) was employed. Genes with a z-score > 1 were defined as overexpressed and <−1 as underexpressed. We discarded all the samples that did not have survival, expression, and subtype data. We worked with a total of 1221 samples for BRCA. For the expression data for COAD and BLCA, RNAseq data of the TCGA were used and the samples were selected under the parameters described above.

We determined the proportion of each BRCA subtype in our sample universe, and the association of the GM-CSF, G-CSF, MCP-1, and RANTES overexpression (z-score > 1) with the overall survival of patients divided into two age groups: ≥55 and <55 years. A data matrix was constructed with the expression data of the four cytokines, overall survival (follow-up of 120 months), and status (censored or dead) of the patients. These data were input to generate Kaplan–Meier curves using the R packages of Survival v3.1-11 and Survminer v0.4.7. The proportion of the samples with overexpression of some of the cytokines in the different subtypes of BRCA was evaluated by PCA and K-means clustering analyses. As input to construct a PCA, we used a list of expression data of each cytokine and subtype data, and we used the ggfortify v0.4.10 in R. After that, cluster v2.0.7-1 was used to perform a K-mean cluster analysis. Finally, we determined the proportion of aggressive BRCA (basal and Her2-enriched) or non-aggressive BRCA (luminal A, luminal B, and normal) samples for each cluster.

A protein–protein interaction network (PPI) was generated using as input GM-CSF, MCP-1 and RANTES in the online platform STRING (https://string-db.org, retrieved August 2020) v11.0. We used an interaction score of 0.900 (highest confidence) and the protein interactions were defined by neighborhood, gene fusion, co-occurrence, co-expression, experimental evidence, databases, and textmining. The PPI was analyzed for centrality measurements (betweenness and closeness centrality) using Cytoscape software v3.6.1. Functional enrichments (biological process and KEGG pathways) analysis was obtained using STRING. We showed the terms with the highest FDR values resulting from the analysis platform. In addition to determining the importance of each node by centrality, we made a gene selection by correlation of expression. We generated a Pearson correlation matrix with the expression data of each node using corrplot v0.84 package in R. Those genes with correlative expression and *p*-value < 0.01 were used to establish the M1-like macrophage extended signature. We determined in the full cohort of 1221 BRCA samples whether the expression of the extended signature was related to other proposed signatures to identify Th1 responses [55], monocyte/macrophage infiltrates [56], immunosuppressive [58], and claudin-low tumors [57]. We downloaded and compared the data of colorectal adenocarcinoma (COAD, *n* = 435, TCGA, PanCancer Atlas) and bladder urothelial carcinoma (BLCA, *n* = 407, TCGA, PanCancer Atlas) databases using cBioPortal. The heatmap (supervised clustering by BRCA subtypes) for BRCA was extracted from cBioPortal; for BLCA and COAD, we downloaded the data of cBioPortal and generated a heatmap in R. We clustered the samples by the average gene expression correlation for each sample. Survival analysis was performed to compare the overall survival of patients with samples clustered by positivity to all the signatures vs. all the remaining samples, or vs. the samples clustered by negativity to the four signatures.

### 4.10. Single-Sample Gene Set Enrichment Analysis

ssGSEA was performed using the GSVA v1.30.0 package in R v3.5.1 with method = ‘ssgsea’. NESs were calculated using as gene sets our M1-like macrophage extended signature, Th1, immunosuppressive, and monocyte/macrophage signature. The analysis was performed for each BRCA subtype. Then, to address the enrichment significance of the extended signature for each BRCA intrinsic subtype, we compared the NES of each subtype with the enrichment score generated after 1000 permutations of randomly sampled gene sets. Gene sets of the same size as the extended signature were used for this analysis within all genes in the array. Additionally, we did a similar analysis performing the permutations only from a list of immune-related genes taken from ConsensusTME [129]. *p*-values were calculated using a Mann–Whitney-Wilcoxon test. We calculated the NES for the extended, immunosuppressive, claudin-low, Th1, and monocyte/macrophage signatures for BLCA and COAD. NES values were compared between the cluster of samples positive to all signatures vs. remaining samples, or vs. the samples clustered by negativity to the four signatures.

### 4.11. Statistical Analysis

GraphPad Prism software v8.3.1. was used for the statistical analysis of the experimental data. For data in which three or more means were compared, we used a Kruskal–Wallis non-parametric test and the Dunnett’s post hoc test for data lacking normality and/or homogeneity. When comparisons were made between two means, the unpaired Student’s t-test was used for normal distribution data and the Mann–Whitney–Wilcoxon test for samples lacking normality and/or homogeneity data. Significant p values were established at ≤0.05 (*), ≤0.01 (**), and ≤0.001 (***) in all statistical analyses. In the overall survival analysis of BRCA public databases, *p*-values were calculated using Log-Rank test and the hazard ratios (HRs) were determined with 95% confidence intervals (95% CIs).

## 5. Conclusions

In this study, we propose an extended gene signature that may explain the polarization of M1-like macrophages in the TME. This extended M1-like signature coexists with Th1 and immunosuppressive signatures, all enriched in claudin-low BRCA samples, and correlating with reduced patient survival. We observed that all these signatures are also present in mesenchymal carcinomas of the colon and bladder, which have been associated with invasive and metastatic features and chemotherapy resistance, and be prone to relapse. Our findings open a window to a better characterization of the claudin-low TME that could lead to better immunotherapy strategies that improve patient prognosis. Finally, our results suggest that macrophages with proinflammatory characteristics also collaborate in the progression and maintenance of the tumor, and not only in antitumor responses as has been usually proposed.

## Data Availability

All data are publics and are available in cBioPortal for Cancer Genomics (www.cBioPortal.org).

## Supplementary Materials

The following are available online at https://www.mdpi.com/article/10.3390/cancers13092248/s1. Figure S1. Four proinflammatory cytokines promote PBM differentiation into M1-like macrophages, Figure S2. T47D cells representative images and vimentin-positive staining, Figure S3. Young patients with breast cancer and overexpression of any of GM-CSF, G-CSF, MCP1, or RANTES cytokines exhibit decreased survival, Figure S4. The extended gene expression signature is concordant with formation of M1-like macrophages and poor survival, Figure S5. Permutation analysis comparing the M1-like macrophage extended signature and random genes related to immunity, Table S1: Samples positive to over-expression of each cytokine (%), Table S2: Summary of overall survival (months) data of patients of the different BRCA intrinsic subtypes that are positive and negative to the extended signature, Table S3: Identity of the genes conforming all signatures and their expected up or down expression.
