# Supplementary material for: Claudin-Low Breast Cancer Inflammatory Signatures Support Polarization of M1-Like Macrophages with Protumoral Activity
Source: Cancers (Basel). 2021 May 7;13(9):2248. doi: 10.3390/cancers13092248 (PMC8125772; doi:10.3390/cancers13092248)
Supplement: Supplementary file 1 [file cancers-13-02248-s001.zip › cancers-1173136-supplementary.pdf]

Supplementary Materials

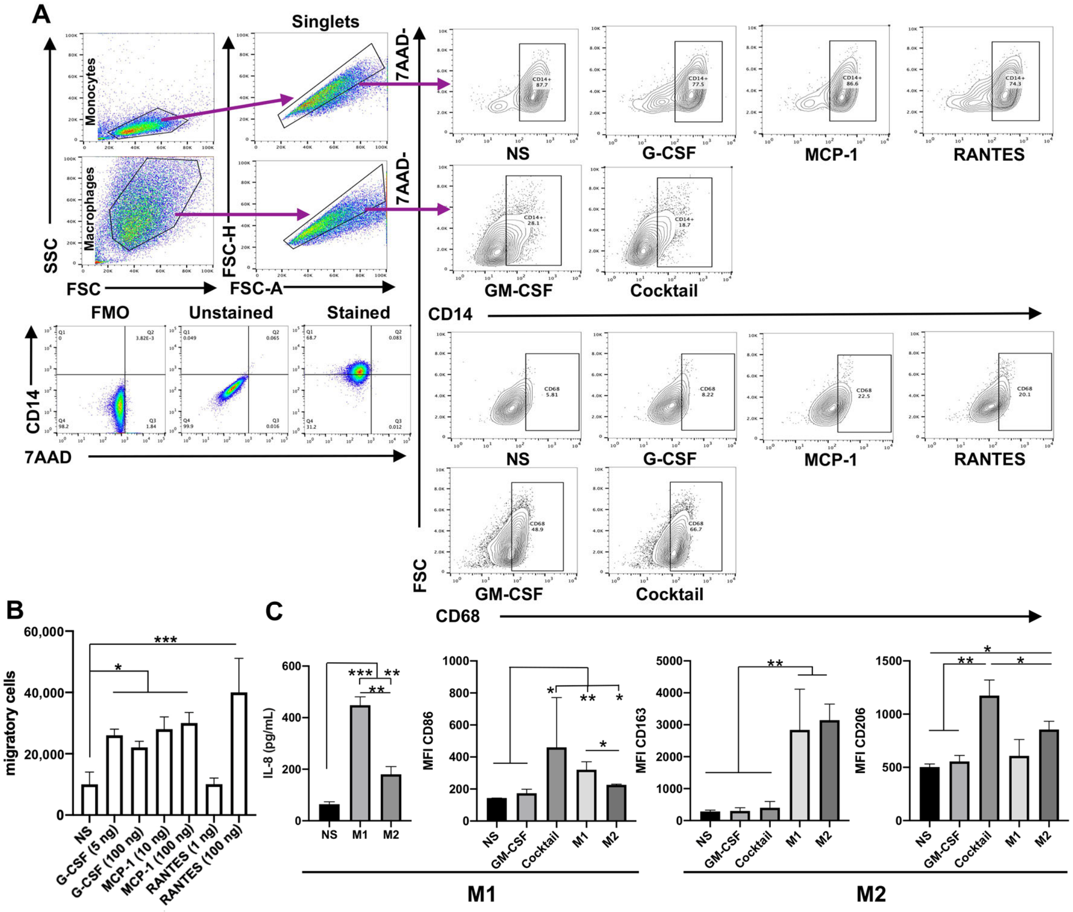

Figure S1. GM-CSF, G-CSF, MCP-1 and RANTES proinflammatory cytokines promote PBM differentiation into M1-like macrophages. (**A**) Flow cytometry of CD14 and CD68. The gates were adjusted according to two populations with different morphological characteristics (size and complexity). The first group is made up of monocytic cells derived from the following conditions: NS and treated with G-CSF, MCP-1 and RANTES individually, and the second group is made of the macrophage-like cells generated with GM-CSF and the cocktail of all cytokines. All the analyzes were performed from live cells (7AAD-). Representative images of one experiment. (**B**) Migration assay of U937 monocytes. We used as chemotactic agent the cytokines at the concentrations found in the cocktail, in addition, we used 100 ng/mL of each cytokine, which is the concentration more often used in other studies. For the statistical analysis all conditions were compared against NS. (**C**) Evaluation of a panel of M1 (IL-8 and CD86) and M2 (CD163 and CD206) macrophage markers. NS: non-stimulated monocytes kept in culture for as many days as the cytokine-induced monocytes. Macrophages induced with either GM-CSF/LPS/IFNγ (M1) or M-CSF/IL-4/IL-13 (M2) were included for comparison. Data represent the mean ± SEM (standard error of the mean) from three independents experiments. Statistical tests were the non-parametric Kruskal–Wallis and Dunnett post-hoc tests, * p <0.05, ** p <0.01 and *** p <0.001.

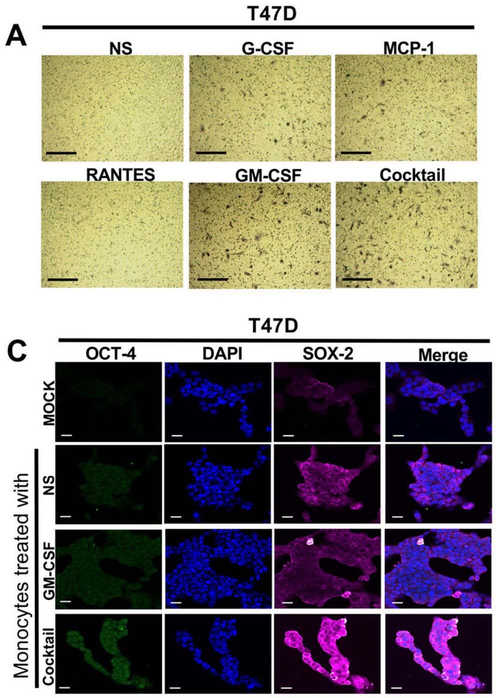

Figure S2. T47D cells representative images and vimentin positive staining. (**A**) Matrigel-based transwell invasion assay. The scale bars indicate 100 μm, magnification 100×. (**B**) Immunofluorescence analysis of epithelial to mesenchymal transition and (**C**) of stemness markers. (**B**) E-cadherin (magenta), vimentin (green) and nuclei (blue); (**C**) OCT4 (green), SOX2 (magenta) and nuclei (blue). (**D**) MDA-MB-231 cells were used as positive control for vimentin staining. The scale bars indicate 20 μm, magnification 60×.

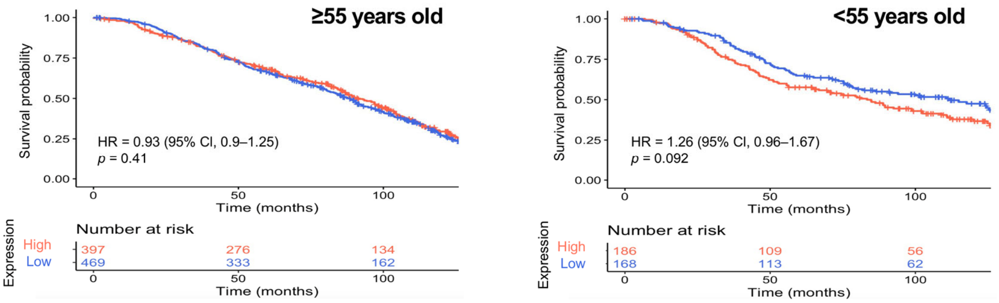

Figure S3. Breast cancer young patients with overexpression of any of GM-CSF, G-CSF, MCP1 or RANTES cytokines exhibit decreased survival. Kaplan-Meier survival curves of breast cancer patients divided by age, ≥55 and <55 years old and comparing GM-CSF, G-CSF, MCP1 or RANTES over-expression (high, z-score >1) against under-expression (low, z-score <-1). P-values were calculated using Log-Rank test and the hazard ratios (HR) were determined with 95% confidence intervals (95% CI).

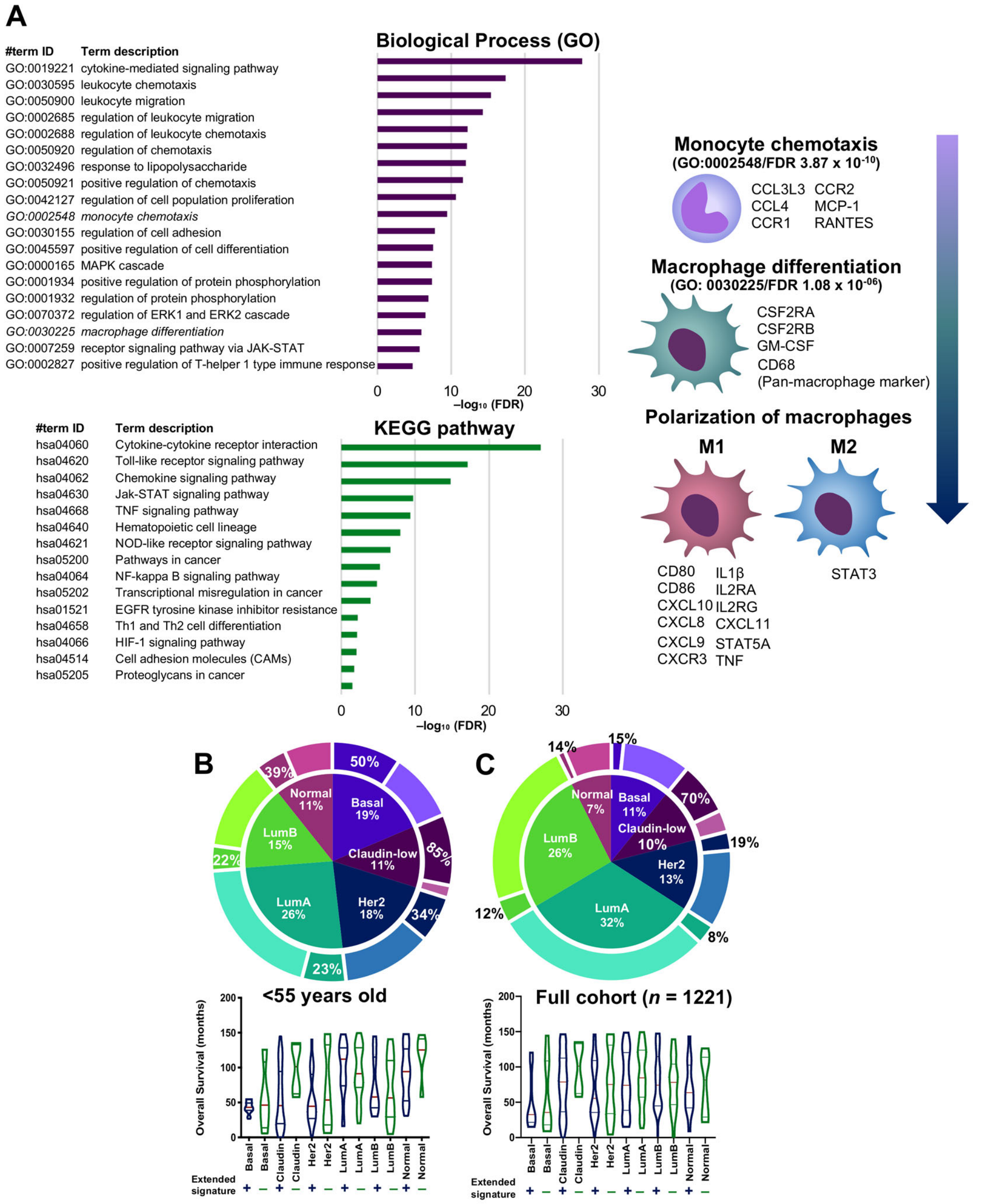

Figure S4. The extended gene expression signature is concordant with formation of M1-like macrophages and poor survival. (**A**) GO and KEGG pathway enrichment analyses with top terms related to the extended gene signature associated with GM-CSF, MCP1 and RANTES. Graphical summary of the top genes along the pathways of monocyte chemoattraction, macrophage maturation and polarization (right). GO term identity and FDR values are provided. Several terms included in the bar graph participate in the polarization of M1 and M2 macrophages, genes included in the signature that participate in this process were taken from the literature, since there is no GO or KEGG term that is specifically assigned. (**B**) Proportion of patients younger than 55 years old divided by subtype (includes claudin-low), outer ring representing in dark colors the proportion of samples positive (one SD above the median value of NES) to the extended signature, light colors represent the rest of the cohort. (**C**) Proportion of the full cohort of patients of METABRIC BRCA database divided by subtype that are positive (one SD above the median value of NES) to the extended signature. Below (**B**) and (**C**) pies, plots with the overall survival of patients with different BRCA intrinsic subtypes and that are positive and negative to the extended signature. Values from plots of (**B**) and (**C**) are shown in Table S2. NES: Normalized Enrichment Score, SD: standard deviation.

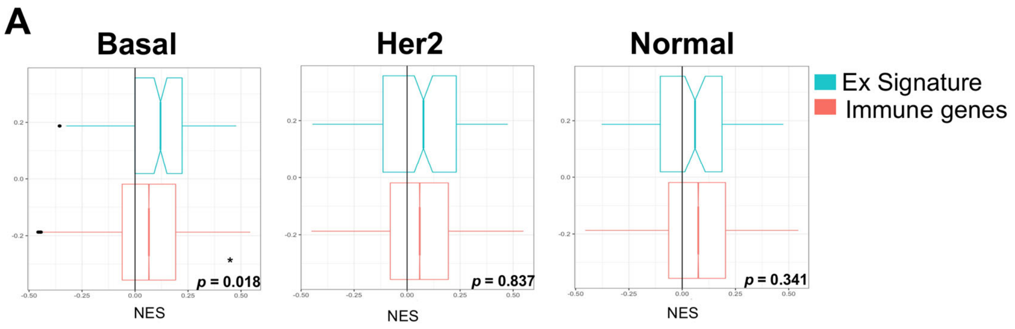

Figure S5. Permutation analysis comparing the M1-like macrophage extended signature and random genes related to immunity (A). Mann-Whitney-Wilcoxon test of NES values for basal, Her2 and normal subtypes. * p <0.05. NES: Normalized Enrichment Score.

Table S1. Samples positive to over-expression of each cytokine (%)

	G-CSF	MCP-1	RANTES	GM-CSF
**Basal**	15.04	22.56	30.08	27.07
**Claudin-low**	15.45	61.79	60.16	28.46
**Her2**	11.88	12.5	25.63	16.88
**LumA**	18.18	7.32	8.08	8.33
**LumB**	16.35	9.75	8.8	14.78
**Normal**	17.58	26.37	20.88	19.78

Table S2. Summary of overall survival (months) data of patients of the different BRCA intrinsic subtypes that are positive and negative to the extended signature.

**Overall Survival (Months)**												
**Extended signature (<55 years)**												
	Basal (+)	Basal (−)	Claudin (+)	Claudin (−)	Her2 (+)	Her2 (−)	LumA (+)	LumA (−)	LumB (+)	LumB (−)	Normal (+)	Normal (−)
n	13	4	34	4	22	10	19	20	12	17	10	5
Med	43.06	46.18	45.61	101.22	44.6	53.55	112	91.27	57.95	56.5	94.23	125.33
Max	54.69	125.7	144.67	135.33	140.33	148.07	147.77	149.6	144.77	140.77	148.03	146.83
Min	26.72	5.83	0.76	57.67	1.77	6.27	16.3	20.27	30.13	4.87	30.97	58
SEM	1.39	25.36	7.59	18.79	7.8	17.32	8.35	7.97	11.72	10.85	9.67	15.5
**Extended signature (full cohort)**												
n	20	8	86	4	31	14	33	59	40	35	13	10
Med	32.58	35.65	78.93	101.22	55.63	75.22	74.1	84.63	74.2	78.16	63.5	81.58
Max	120.43	144.43	146.37	135.33	146.06	146.4	148.8	149.43	147.36	139.3	143.16	126.4
Min	15.3	9.13	0.77	57.67	1.77	4.43	15.2	13.4	2.53	1.27	9.06	21.9
SEM	8.3	18.32	4.53	18.79	7.43	13.33	7.49	5.19	6.18	6.28	10.27	13.41

Sample size (n), median (Med), maximum (Max), minimum (Min) and standard error of the mean (SEM). For this analysis, positive samples were considered those 1 standard deviation (SD) above the median of the NES (normalized enrichment score) value, while negative samples were 1 SD below the NES.

Table S3. Identity of the genes conforming all signatures and their expected up or down expression.

**Extended signature**				**Th1**		**Monocyte/macrophages**		**Claudin-low**		**Immunosuppressive**	
Gene	Expected regulation	Gene	Expected regulation	Gene	Expected regulation	Gene	Expected regulation	Gene	Expected regulation	Gene	Expected regulation
CCL3L3	UP	IL2RA	UP	CD4	UP	FCN1	UP	EPCAM	DOWN	CD274	UP
CCL4	UP	IL2RG	UP	DPP4	UP	VCAN	UP	CLDN3	DOWN	IDO1	UP
CCR1	UP	IL6	UP	HAVCR2	UP	CD14	UP	CLDN4	DOWN	FASLG	UP
CCR2	UP	MCP1	UP	IFNA1	UP	CD33	UP	CLDN7	DOWN	CTLA4	UP
CD68	UP	***RANTES***	UP	IFNGR1	UP	CSF3R	UP	CDH1	DOWN	PDCD1	UP
CD80	UP	***STAT3***	DOWN	IL2	UP	***CD68***	UP	SNAI1	UP	LAG3	UP
CD86	UP	STAT5A	UP	KLRD1	UP	CD80	UP	MUC1	DOWN	PDCD1LG2	UP
CSF2RA	UP	***TNF***	UP	TNFSF11	UP	CD86	UP	TWIST2	UP	IL10	UP
CSF2RB	UP			IL-12A	UP	CXCR3	UP	MME	UP	TGFB1	UP
**CXCL11**	UP			CCR1	UP	GM-CSF	UP	ALDH1A1	UP	PTGS2	UP
**CXCL10**	UP			***GM-CSF***	UP	***RANTES***	UP	ZEB2	UP		
CXCL8	UP			IL10RA	UP	***TNF***	UP	TWIST1	UP		
**CXCL9**	UP			***RANTES***	UP	***STAT3***	DOWN	ITGB1	UP		
CXCR3	UP			***TNF***	UP			SNAI2	UP		
GM-CSF	UP							ZEB1	UP		
IL10RA	UP							THY1	UP		
IL1B	UP							VIM	UP		

Genes in bold are genes shared by signatures
